# Defying convention in the time of COVID-19: Insights into the role of γδ T cells

**DOI:** 10.3389/fimmu.2022.819574

**Published:** 2022-08-11

**Authors:** Marta Sanz, Brendan T. Mann, Alisha Chitrakar, Natalia Soriano-Sarabia

**Affiliations:** Department of Microbiology, Immunology and Tropical Medicine, George Washington University, Washington, DC, United States

**Keywords:** gamma delta (γδ) T cells, SARS-CoV-2, innate immunity, aminobisphosphonates COVID-19, COVID-19

## Abstract

Coronavirus disease 2019 (COVID-19) is caused by severe acute respiratory syndrome coronavirus 2 (SARS-CoV-2). COVID-19 is a complex disease which immune response can be more or less potent. In severe cases, patients might experience a cytokine storm that compromises their vital functions and impedes clearance of the infection. Gamma delta (γδ) T lymphocytes have a critical role initiating innate immunity and shaping adaptive immune responses, and they are recognized for their contribution to tumor surveillance, fighting infectious diseases, and autoimmunity. γδ T cells exist as both circulating T lymphocytes and as resident cells in different mucosal tissues, including the lungs and their critical role in other respiratory viral infections has been demonstrated. In the context of SARS-CoV-2 infection, γδ T cell responses are understudied. This review summarizes the findings on the antiviral role of γδ T cells in COVID-19, providing insight into how they may contribute to the control of infection in the mild/moderate clinical outcome.

## Introduction

### The beginning of the pandemic

Beginning December 2019, there was a surge of pneumonia cases of unknown etiology in Wuhan City, Hubei Province, Central China. Genomic sequencing (1) showed that this pneumonia was caused by a novel coronavirus (CoV) that belonged to the B lineage of the Beta-CoV genus along with SARS-CoV and MERS-CoV (1). Its similarity to the original SARS-CoV sequence led to the naming severe acute respiratory syndrome coronavirus 2 (SARS-CoV-2) and the associated illness was named coronavirus disease 19 (COVID-19). CoVs belong to the family *Coronaviridae* (subfamily *Coronavirinae*) and can infect a wide range of animal hosts. In humans, CoV-induced diseases, such as SARS, MERS, and currently COVID-19 (2, 3), can range in severity from a common cold to the fatal acute respiratory distress syndrome (ARDS). The origin of SARS-CoV-2 and the first transmission event of the disease is still unclear. However, initial group analysis pinpointed the source of the outbreak to a seafood market in Wuhan. This market is known for selling exotic animals for human consumption and it is postulated that this could be the point where the zoonotic transmission occurred (3). The route of transmission between humans is through contact with the nasopharyngeal secretions, including saliva of infected people. Transmission occurs mainly by direct contact with respiratory droplets but also hands or objects contaminated with these secretions (4, 5).

### SARS-CoV-2 structure and life cycle

**Figure 1:**
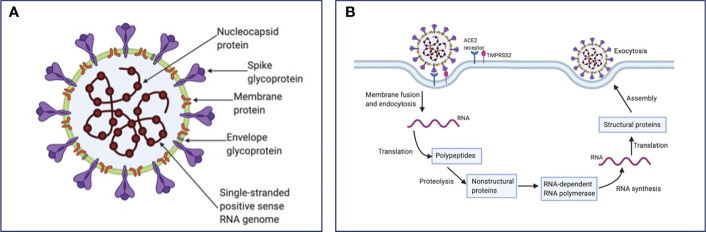
SARS-CoV-2 structure and life cycle. **(A)** SARS-CoV-2 contains four structural proteins: spike surface glycoprotein, membrane, nucleocapsid and envelope protein, as well as eight accessory proteins (not represented). **(B)** Coronavirus particles bind to cellular attachment factors and specific S interactions with the cellular receptors ACE2 and TMPRSS2, promoting viral uptake and membrane fusion. Positive sense single-stranded RNA is released, partially translated into SARS-CoV-2 polymerase protein, and transcribed. Structural proteins and accessory proteins (N, S, M, and E) results after RNA subgenomic translation, that are inserted into the ER–Golgi compartment for virion assembly. Subsequent positive-sense RNA genomes are incorporated into newly virions, which are secreted from the plasma membrane.

SARS-CoV-2 consists of a positive-sense single-stranded RNA genome, approximately 30 kb in length (6). The overall structure of the virus includes the viral genome contained within a nucleocapsid and encased with a glycosylated envelope. Of note, the nucleocapsid is arranged in a helical symmetry, which is not a common characteristic of positive-sense RNA viruses (7). The main structural proteins of coronaviruses are described in detail below (**Figure 1A**). (1) Spike (S) Glycoprotein is a multifunctional class I transmembrane protein and varies in size from 1160 to 1400 amino acids. It is located on the surface of the virion, giving the virus its characteristic crown appearance. Foe the entry of virion particles into the cell, the S glycoprotein is required through its union with different host cell receptors (8).. (2) Membrane (M) protein is the most abundant structural protein in the virion, giving a definite shape to the viral envelope. M protein is related to the shape and size of the virus (9). It binds to the nucleocapsid and plays the role of the central organizer during viral assembly. (3) Nucleocapsid (N) protein plays an important role in the packaging of viral RNA into ribonucleocapsid. This protein mediates viral assembly by interacting with the viral genome and the M protein, facilitating transcription and replication of viral RNA (10, 11). (4) Envelope (E) protein has different functions involved in the entry, assembly, and release of the virus (12, 13). It is also known to act as a viroporin which integrates into the host membrane and alters the flow of ions such as Na^+^ (14, 15).

As recently reviewed, SARS-CoV-2 enters host cells by the interaction of its surface Spike (S) protein with the host’s angiotensin-converting enzyme 2 (ACE2) receptor, a membrane exopeptidase primarily expressed in the kidney, lungs, and heart (16). However, this process also requires a cellular protease that helps the cleavage of the spike protein and fusion of the cell and viral membranes. This cellular transmembrane protease is TMPRSS2 (17). Both of these proteins, TMPRSS2 and ACE2, determines viral entry (18, 19). After the virus is endocytosed, the nucleocapsid containing the RNA genome, is released into the cytoplasm of the cell. Parts of the endoplasmic reticulum are then appropriated to form double membraned vesicles which protect the viral genome to ensure replication (20). The viral genome gets translated by using the protein translation machinery of the host. Two viral proteases are used for cleavage of proteins into structural and nonstructural viral proteins. Viral particles are assembled in the endoplasmic reticulum/Golgi compartment and infectious virions are released from the cells through exocytosis (21–23). SARS-CoV-2 life cycle is summarized in **Figure 1B**.

## SARS-CoV-2 disease: COVID-19

### Treatment and vaccines

COVID-19 has emerged as a complex disease affecting many body systems and generating a wide spectrum of clinical manifestations. Understanding SARS-CoV-2 immunopathogenesis is key to developing effective treatments. Currently, there are several drugs for the treatment of COVID approved by the FDA including several first line antivirals used to treat other infections. The most widely used are described briefly. Remdesivir, a broad-spectrum antiviral with established activity against several viruses such as respiratory syncytial virus (RSV), Ebola virus, MERS-CoV and SARS-CoV (24, 25) is also capable of inhibiting the replication of SARS-CoV-2 (26, 27). It is one of the first line drugs recommended for use in patients who require hospitalization and the support of supplemental oxygen (28). Lopinavir, an antiretroviral of the protease inhibitor class used for HIV treatment, is capable of disrupting viral replication and RNA release from host cells by inhibiting proteases such as 3CLpro, the main SARS-CoV-2 protease critical for viral replication (29). The combination of Lopinavir with IFN-1β significantly reduced the time to clinical improvement in patients with moderate symptoms (30) although this benefit did not extend to patients with severe symptoms (31).

As a result of an incredible scientific effort, we have seen the development of several highly effective vaccines in a short period of time. COVID-19 vaccines can be divided into three categories: (1) Inactivated vaccines, protein-based vaccines consisting of virus particles that generate target antigens *in vitro*, (2) virus-vectored, DNA or mRNA vaccines, are gene-based vaccines that encode proteins of the pathogen are delivered and (3) live-attenuated virus vaccines, it is a combination of protein-based and gene-based to produce protein antigen or antigens *in vitro* and *in vivo* (32–34). Despite the efficacy of these vaccines, we still do not fully understand the role of innate and adaptive immunity against SARS-CoV-2. Specifically, this article aims to give a comprehensive review of available literature covering the role of innate-like γδ T cells fighting SARS-CoV-2 infection.

### SARS-CoV-2 infection can induce a cytokine storm

A cytokine storm is defined by the rapid proliferation and hyperactivation of T cells, macrophages and Natural killer (NK) cells resulting in excess secretion of inflammatory cytokines and chemical mediators released by both immune or nonimmune cells (35). In older adults, having an unbalanced pro-inflammatory environment can further enhance the inflammatory responses to SARS-CoV-2 infection, leading to an exacerbated cytokine storm and could also influence ACE2 expression facilitating viral entry (36). This cytokine production leads to positive feedback on other immune cells by recruiting them to the area of inflammation causing an exponential increase in inflammation and tissue damage (37). The main cytokines involved in the cytokine storm are interleukins (IL), interferons (IFN), tumor necrosis factor (TNF), colony stimulating factors (CSF), chemokines, and growth factors (GF) (35). The induction of cytokine production typically occurs following viral entry. Target cells such as respiratory epithelial cells, alveolar cells, macrophages, and blood circulating monocytes are activated through pattern recognition receptors (PRRs) that lead to stimulation of the pro-inflammatory NF-κB cascade (38, 39). In addition, viral RNA enters endosomes and activates intracellular toll-like receptors (TLRs), mainly TLR7/8, inducing the expression of numerous inflammatory factors, cytokines, and chemokines (38, 40). One of the most detrimental effects caused by the cytokine storm is acute lung injury (35).

Recent studies have shown that patients with severe disease had high concentrations of pro-inflammatory cytokines (IL-1β, IFN-γ, interferon gamma inducible protein-10, IP-10, and monocyte chemoattractant protein-1, MCP-1) which were associated with pulmonary inflammation and extensive lung damage (41). Similarly, severe cases often display heightened activated T-helper-1 (Th1) cell responses including increased production of IFN-γ, TNF-α, and IL-2 as well as macrophage overactivation (42), both of which contribute to increased inflammation and tissue damage. The observation that patients admitted to the ICU had higher granulocyte-colony stimulating factor (G-CSF), IP-10, MCP-1, MIP-1α, and TNF-α concentrations, further suggests that the development of a cytokine storm is associated with disease severity. However, an increase in T-helper-2 (Th-2) associated cytokines IL-4 and IL-10 has also been reported in severely infected patients. These findings are consistent with other studies in which severe COVID-19 patients had mixed high levels of cytokines (IL-2, IL-6, IL-7, IL-10, IP-10, MCP-1, TNF-α) than patients with mild and moderate infections (43, 44). Therefore, an adequate Th-1/Th-2 balance plays an important role controlling the severity of the disease. However, more studies are needed to characterize the Th1 and Th2 responses, as well as when and how dysregulation starts impacting the clinical outcome of the disease.

The prolonged release of inflammatory cytokines induces a feedback loop of subsequent cytokine production that can lead to cell and organ damage. This cytokine storm can be caused by an aberrant host response to infection provoking a severe clinical syndrome known as sepsis, (45). Critically ill COVID-19 patients may develop organ dysfunction due to a dysregulated response to infection and develop sepsis (46, 47). Criteria for the diagnosis and treatment of bacterial sepsis is analogous to SARS-CoV-2 infection and has been applied to understand severe COVID-19 (48). However, there are some differences between sepsis caused by SARS-CoV-2 and bacterial sepsis. A retrospective study investigated whether there are differences in the immune system status of these two types of sepsis. A total of 64 bacterial sepsis patients and 43 patients with SARS-CoV-2 sepsis were included (49). The results of this study showed that there are key differences between the two types of sepsis, such as a milder cytokine storm in SARS-CoV-2 sepsis as well as higher immunoglobulin and complement protein levels. However, neutrophil, monocyte, infection biomarkers, individual lymphocyte subset counts (total T lymphocyte, CD4+ T cell, CD8+ T cell, B cell, and NK cell counts), and lymphocyte subset functions were similar in bacterial sepsis patients and SARS-CoV-2 sepsis patients. Treatment protocols for sepsis have a well-established history in most healthcare systems. Considering the similarities in both the immunopathogenesis and pathophysiological manifestations, our knowledge of sepsis could inform the management of severity COVID-19 (50).

### Innate and innate-like immune responses to SARS-CoV-2 infection

**Figure 2:**
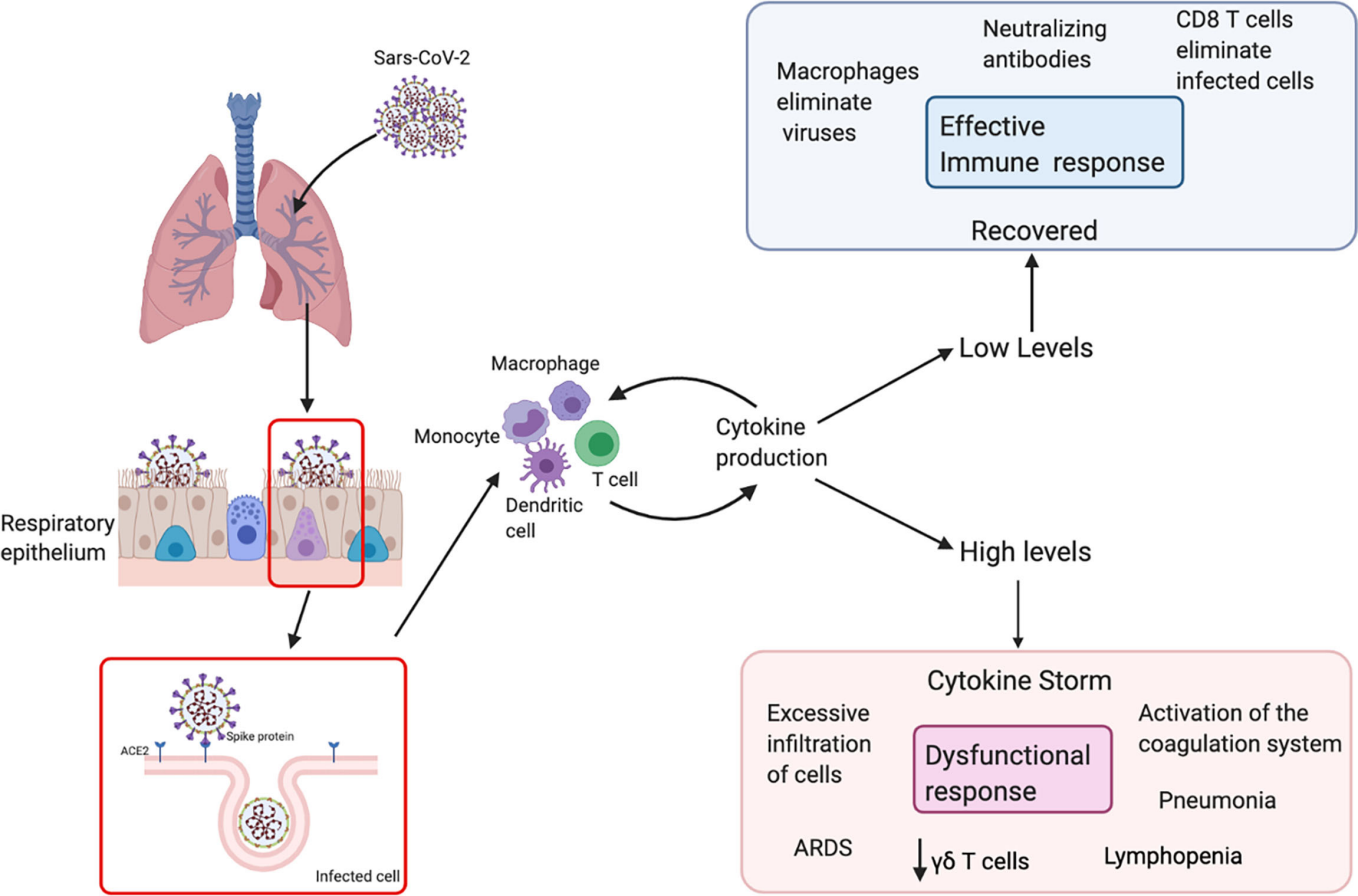
Schematic immune responses to SARS-CoV-2. The SARS-CoV-2 virus recognizes ACE-2 in respiratory epithelial cells, facilitating viral entry. After viral replication, the virus is released and recognized by the immune system. T cells and dendritic cells are activated through pattern recognition receptors. The virus induces the expression of numerous inflammatory factors, the synthesis of type I interferons and production of cytokines. High levels of cytokines cause a cytokine storm, which leads to a dysfunctional response with an excessive infiltration of cells, decrease in different populations of T cells in the blood such as γδ T cells and surviving T cells are immunologically exhausted, this provokes progression to acute respiratory distress syndrome (ARDS).

The clinical course of COVID-19 is very similar to other respiratory infections such that 80% of patients present mild to moderate symptoms (**Figure 2**). SARS-CoV-2 primarily targets cells in the upper and lower respiratory tract, as well as pulmonary cells (51). Macrophages and NK cells belong to the innate immune system and constitute one of the first defense mechanisms (52). Pathogens are removed from circulation by phagocytosis, and processed antigens are displayed to initiate adaptive immune responses. This includes the production and secretion of inflammatory cytokines and chemokines that recruit various immune cells to the site of infection and regulate T cell responses to the pathogen (53, 54). γδ T cells, which are generally classified as innate-like T cells, secrete several cytokines including IFN-γ and TNF-α and also cytotoxic components such as perforin and granzymes (55).

γδ T cell responses to coronavirus infections have been understudied, with a previous report on SARS-CoV infection showing a potent cytolytic activity against infected target monocytic cell lines (56). Although more intense research has been performed in other airway infections such as influenza, their role in SARS-CoV-2 infection remains understudied.

## γδ T cell subpopulations and ligands

γδ T cells’ unique properties enable them to have a critical tole linking innate and adaptive immune responses (57, 58). These cells are key contributors to pulmonary mucosal immunity including tissue repair and rapid response against numerous respiratory pathogens (59–61), and therefore their potential role fighting SARS-CoV-2 infection is worthy of investigation.

γδ T cells are recognized for their contribution to tumor surveillance, fight against infectious diseases, and autoimmunity (57, 58, 62). In contrast to conventional αβ T cells, the T-cell receptor (TCR) of γδ T cells is comprised of variable γ and δ chains that recognize non-peptide antigens in the absence of MHC molecules. γδ T cells elicit non-redundant functions when compared to conventional αβ T cells (57, 58). Within circulating human T cells, γδ T cells account for 0.5–10% and are classified into two major peripheral blood subpopulations based on the δ chain usage, Vδ1 and Vδ2 T cells (57). The Vδ2 chain is present in the majority of circulating γδ T cells and is almost always paired with the Vγ9 chain (Vγ9Vδ2 T cells) (63–65). Vδ2 T cells specifically recognize low-weight phosphorylated metabolites of isoprenoid biosynthesis compounds, or mevalonate pathway, referred to as phosphoantigens (P-Ags) (66, 67). This pathway is essential for the synthesis of cholesterol and precursors for prenylation of proteins and as such are produced by both eukaryotes and prokaryotes and can therefore act as self (host) or non-self (pathogen) antigens. The most potent, naturally occurring P-Ag known to date is (E)-4-Hydroxy-3-methyl-but-2-enyl pyrophosphate (HMB-PP), an intermediate of the MEP (non-mevalonate) pathway utilized by several pathogenic bacterial species (68). Another, less potent activator is isopentenyl pyrophosphate (IPP), an intermediate of the mevalonate pathway that increases in response to cellular stress and is present both in eukaryotic and procaryotic cells (69, 70). Aminobisphosphonates (N-BPs) bears structural similarity to pyrophosphates and specifically activate Vδ2 T cells. N-BPs inhibit one key enzyme of the mevalonate pathway, farnesyl pyrophosphate synthase, leading to an accumulation of IPP (71), that is then presented to Vδ2 T cells independent of major histocompatibility (MHC) molecules, but in the context of butyrophilin (BTN) molecules (72, 73). A recent study identified that in addition to BTN3A1, BTN2A1 plays an important role as a ligand that binds to the Vγ9 TCR γ chain (72). It is believed that once p-Ag binds to BTN3A1 through its intracellular domain, the BTN2A1–BTN3A1 complex engages the γδ TCR *via* two distinct binding sites: BTN2A1 binds to Vγ9 region, whereas another ligand (possibly BTN3A1) binds to the opposing Vγ9 and Vδ2 chains. Besides detecting p-Ags, γδ T cells can recognize cells through molecules that are expressed at the cell surface in a stress-induced manner. For example, endogenous proteins, such heat shock protein 60 (74, 75) or FI-ATPase (76), that can be ectopically expressed on the cell membrane upon transformation and recognized by Vδ2 TCRs to promote tumor cell lysis. In addition, γδ T cells recognize malignant cells through the engagement of innate-like activating receptors like NKG2D, and natural cytotoxicity receptors (NCRs) (57, 77). NKG2D acts as a costimulatory signal upon recognition of stress markers MHC class I chain related protein A and B (MICA, MICB), and UL16 binding proteins (ULBPs). Activation through this pathway provokes the secretion of proinflammatory cytokines TNF-α and IFN-γ as well as direct cytolytic activity mediated by the release of perforins and granzymes (78–80). On the contrary, the natural antigen for Vδ1 T cells has yet to be defined. These cells are mainly found within the tissue where they have a critical role in immunosurveillance (57).

### Functional plasticity of γδ T cells

γδ T cells exhibit a high degree of polyfunctionality, or functional plasticity as regulatory and effector cells, as characterized by their membrane receptors and cytokine production (57). γδ T cells can secrete a range of different cytokines and can directly interact with other immune cell populations (81). Distinct subpopulations of γδ T cells are capable of producing either anti-inflammatory or pro-inflammatory signals, giving them a direct role in maintaining immunological homeostasis and response to disease (82–84). They exert different immunomodulatory and adjuvant functions on CD4 T, CD8 T, B, and dendritic cells (DC) which vary according to the infectious challenge or environment, and the subset of γδ T cells involved in the antiviral response (85). γδ T cells execute direct cytolytic activity mediated by the release of perforin and several types of granzymes (86). γδ T cells may also induce apoptosis through the expression of tumor necrosis factor receptor superfamily members TNF- related apoptosis-inducing ligand (TRAIL) or FasL (87, 88). Both of which have been demonstrated to be important for mediating the resolution of inflammation and clearing tumor cells. Effector γδ T cells also express CD16 (FcγRIII) which affords them the ability to execute antibody-dependent cellular cytotoxicity (ADCC) against virally infected cells as well as phagocytosis of opsonized cell-free pathogens (89, 90). Studies from our group have highlighted the importance of the CD16 receptor expression as a marker of cytotoxic capacity associated to Vδ2 T cell elimination of HIV-latently infected CD4 T cells (91, 92).

### γδ T cells in viral infections

γδ T cells play a key role in controlling a variety of different viral infections. Their anti-HIV activity was recognized since the beginning of the pandemic (93) and although not completely yet understood, their key role fighting the infection has been documented (86, 94). Less is known about their specific involvement in HIV latency, which is the focus of our studies (86, 91, 92, 94). In respiratory infections, such as influenza or respiratory syncytial virus (RSV), lung-resident and infiltrating γδ T cells play critical roles in controlling the severity of the infection (89, 90, 95, 96). More intensive research has been performed in influenza, providing important insights on general γδ T cell responses to viral infections that cause respiratory distress. Using a murine model of influenza, it was recently reported that early production of IL-17 by γδ T cells promoted viral clearance (89) and more importantly, adoptive cell transfer of expanded γδ T cells resulted in cells trafficking to the lungs and induction of potent antiviral function that led to control of viral replication (90). In line with these results, activated γδ T cells express high levels of chemokine receptors CXCR5, CCR1 and CCR5 allowing their migration along CCL3 and CCL5 chemotactic signals towards the lungs (97, 98). Interestingly, influenza-infected macrophages and DCs alter the mevalonate pathway leading to the production of IPP and subsequent γδ T cell activation directly conferring immune protection independent of viral subtypes (99).

## γδ T cell responses to SARS-CoV-2

Lymphopenia is associated with disease progression where a significant reduction in T cell counts is often observed in severe COVID-19 patients compared to non-infected and mild cases (100, 101). SARS-CoV-2 has the ability to evade the immune system, allowing the virus to proliferate and disseminate into various tissues. Dysregulation of host immunity has been linked to the severity of the disease. This includes macrophage hyperactivation, the development of a cytokine storm, as well as the depletion of key lymphocytic populations such as NK cells and cytotoxic T cell lymphocytes (CTLs) cells (35).

γδ T cell frequency in the blood of hospitalized COVID-19 patients is lower compared to healthy controls (102–104), indicating γδ T cells as one of the most affected lymphocyte subsets (105). Carissimo et al. (105) evaluated the frequency of different T cell populations, CD8, CD4, γδ (Vδ1 and Vδ2), and mucosal-associated invariant T (MAIT) cells in the acute phase of the infection in COVID-19 patients showing a decrease in cytolytic populations CD8, MAIT and Vδ2 T cells. On the contrary, Vδ1 and CD4 T cells did not show a significant decrease during infection. In addition, as the disease severity increased, there was a gradual reduction of Vδ2 T cells in the peripheral blood, which was hypothesized to be a consequence of activation and infiltration of these cells into the lungs (105). This inverse correlation was more pronounced in Vδ2 and CD8 T cells, which suggested that there was a selective activation and infiltration of these cells in the lungs. In line with these observations, a recent work on the first lung transplant of a COVID-19 patient, highlighted a key role of γδ T cells being specifically recruited to the lungs (106). In addition to decreased circulating Vδ2 T cells, other studies reported a switch to an effector memory population at the time of hospital admission compared to healthy control (102, 107, 108). This increase in memory phenotype provides further insights into the possibility of effector-like γδ T cells recruitment from the blood to the lungs therefore, strengthening their involvement in the immune response against SARS-CoV-2 (109).

γδ T cells from COVID-19 patients with mild symptoms exhibited a strong activated phenotype based on CD25 expression, although early activation marker CD69 and exhaustion marker PD-1 levels were similarly expressed in healthy donors (108). This limited data suggests that γδ T cells were not exhausted, raising the possibility that CD69 was expressed earlier during infection followed by reversion to the quiescent state during prolonged recovery. Whether this is the case for severe cases of COVID-19 requires further investigation. In addition, patients in the acute phase of infection had elevated expression of CD38 in Vδ2 T cells compared to healthy individuals. Interestingly, Carissimo et al. also found that the immature neutrophil-to-Vδ2 T cell ratio was an excellent tool to predict patient progression to pneumonia (105).

Some γδ T cell subsets constitutively express CD8 while CD4 is generally absent (110). However, we and others previously reported that they can transiently upregulate CD4 expression upon activation (111, 112). Similarly, the proportion of γδ T cells expressing CD4 was increased in COVID-19 compared to healthy donors, while frequency of CD8 γδ T cells remained unchanged (108). In our previous study, CD4 was transiently upregulated after *in vitro* exposure to IL-2, without requiring TCR-antigen recognition suggesting that CD4 expression on Vδ2 cells could constitute an additional activation marker (111). Interestingly, a higher frequency of CD16 expression was observed in moderate COVID-19 disease and was almost absent in the severe cases of the disease (113, 114). Therefore, similar to HIV infection, in addition to its involvement in ADCC, CD16 could constitute a critical marker to determine the extent of the cytotoxic capacity of γδ T cells and their ability to control SARS-CoV-2 infection (115). The involvement of γδ T cell-mediated ADCC in COVID-19 responses is currently unknown, and constitute an additional area of critical investigation, similar to NK-ADCC mediated functions (116, 117).

Due to the dearth of studies that specifically focus on γδ T cell responses to COVID-19, there are important caveats to consider when comparing these collective findings. Patient demographics (e.g., age, biological sex, and race) can influence γδ T cell frequencies, phenotypes, and functions during both normal health and disease (118, 119). Since age is an additional risk factor for severe COVID-19 disease and Vδ2 T cell frequencies are diminished in the elderly where general a systemic low-grade inflammation is present, the low Vδ2 T cell count could explain why the disease is more severe in older patients (120, 121). The lack of age and sex-matched cohorts within most existing studies presents a clear limitation that should be addressed in future investigations. There is also a growing body of evidence suggesting COVID-19 disease severity is associated with pre-existing comorbidities such as obesity and Type 2 diabetes. These chronic inflammatory health conditions may independently contribute to alterations in γδ T cells including their antiviral responses (122, 123). This necessitates mechanistic studies to determine the independent contribution of SARS-CoV-2 infection on γδ T cell characteristics. Lastly, the definition and stratification of disease severity is often discordant between studies. Identifying robust clinical biomarkers as well as widespread utilization of nucleic acid and rapid antigen testing may allow for more clearly defined methods for analyzing γδ T cells at different stages of COVID-19.

## Immunotherapy for infectious diseases

Few studies for the therapeutic use of Vδ2 T cells to treat infectious disease have been performed to date. A pilot study analyzed the effect of combined treatment with the N-BP zoledronate and interleukin-2 in people living with HIV without antiretroviral treatment (124). The authors observed a partial restoration of Vδ2 T cell functionality including enhancement of dendritic maturation and possibly HIV-specific CD8 lymphocyte anti-viral responses.

A therapeutic effect against influenza has been demonstrated (125), showing that human N-BP expanded Vδ2 T cells killed influenza virus-infected cells and inhibit viral replication *in vitro* (90). Further work demonstrated in immunodeficient mice reconstituted with human peripheral mononuclear cells, that the N-BP pamidronate reduced disease severity caused by human seasonal H1N1 and avian H5N1 influenza virus. In a more recent study, the potential of pamidronate on treating H7N9 virus-infected humanized mice was evaluated showing that intraperitoneal injection of the drug induced expansion of Vδ2 T cells that controlled both viral replication and inflammation in affected lungs (126). The therapeutic effect of Vδ2 T cells has also been demonstrated in other infections such as flavivirus infections (127, 128) and Hepatitis C virus (129, 130).

The tuberculosis vaccine, Bacille Calmette-Guerin (BCG), also affords protection against other diseases, due to immune mechanisms such as activation of non-conventional T-cells, and cross-reactive adaptive immunity (131–133). A recent study used a rhesus macaque model to explore the protective efficacy of aerosol-delivered BCG vaccination against SARS-CoV-2 (134). Immune parameters were monitored in vaccinated and unvaccinated rhesus macaques for 28 days following aerosol BCG vaccination. Interestingly, CD14+ monocytes and Vδ2 T cell frequencies increased rapidly in vaccinated animals following SARS-CoV-2 infection. Despite the lack of efficacy of the vaccination clearing infection, this study adds on the potential immunotherapeutic role of γδ T cells for SARS-CoV2.

These findings provide a strong justification for further studies aimed at translating Vδ2 T cells into the clinic for infectious diseases that currently have limited curative treatment options.

## Conclusions and future perspectives

Despite the limited studies on γδ T cells, there is evidence that indicates their active participation fighting SARS-CoV-2 infection similar to their known pivotal antiviral and cytotoxic functions against other viral infections. In addition to their migration and infiltration capabilities into sites of infection, including the lungs (106), their functional plasticity is reflected in their ability to secrete numerous cytokines and crosstalk with other cells including monocytes/macrophages and DCs, to induce strong adaptive immune responses. Our capacity to manipulate the mevalonate pathway allows us to generate γδ T cells with enhanced migration and cytotoxic capabilities (135) and as such, γδ T cell immunotherapy is being pursued from several angles to treat different malignancies (136, 137), and for HIV cure (91, 92). The use of nBPs to prevent or treat COVID-19 was previously postulated (138) although a recent study showed that oral nBPs did not prevent, nor ameliorate severe COVID-19 disease (139). One possible explanation for this outcome could be related to Vδ2 T cell exhaustion after repeated dosage of the nBP (140). As recently reviewed, nBPs could be beneficial for SARS-CoV-2 acute response in the lung (141) since nBPs directly activate Vδ2 T cell homing to tissues (105, 142) and therefore, migration into the lungs in response to SARS-CoV-2 would be achieved. As we reviewed herein, Vδ2 T cells exhibit a potent adjuvant function that includes modulation of B, T, DC, and NK cells functions (143) and their specific activation could shape the full adaptive immune response.

An alternative approach to modulating the mevalonate pathway are statins. Statins are widely used for the treatment of hypercholesterolemia and they function by inhibiting HMG-CoA reductase, which is the rate-limiting enzyme in the mevalonate pathway (144, 145). SARS-CoV-2, like other enveloped viruses, depend on their lipid envelopes for entry and replication in host cells (146). Therefore, to reduce entry of coronaviruses, cholesterol can be depleted from the plasma membranes of target cells (147) In addition, statins have anti-inflammatory and immunomodulatory effects, inhibiting the secretion of proinflammatory cytokines (148) and suppressing T cell activation (149). Different observational and experimental studies suggest that treatment with statins could be associated with better prognosis in severe COVID-19 infection. A meta-analysis demonstrated that the use of statins was associated with a lower risk of mortality in COVID-19 patients (150). However, more investigation is still needed to confirm these findings.

The major adverse effect of intravenously administered nBPs is the acute phase response associated with the release of TNF-α and IL-6 (151–153). Combined administration of nBPs and statins could be a possibility for SARS-CoV-2, similar to a pilot study in patients with (bone)metastasized malignancies receiving nBP-treatment (154). Although statins reduced the production of perforin and GrzB from Vδ2 T cells, they did not prevent nBP effects increasing cell frequency, and IFN-γ and TNF-α production. An additional study showed that prior use of statins avoided the acute phase response by inhibiting nBP-induced production of IL-6 (155). The sequential use of statins and nBPs in strategic paucity to treat mild versus severe COVID-19, constitutes a valuable area of research to find the balance between immune hyperactivation and suppression.

## Concluding remarks

Although SARS-CoV-2 infection research has been centered in other more abundant T cell populations, available studies demonstrate a critical involvement of γδ T cells in fighting SARS-CoV-2 infection, and in determining the severity of COVID-19. Exploiting Vδ2 T cell functional plasticity could lead to better means of prevention and mitigation of severe cases, potentially improving clinical outcomes.

## Author contributions

Writing original draft, MS, AC, BTM., and NS-S. Resources, NS-S. Conceptualization, NS-S. All authors have read and agreed to the published version of the manuscript.

## Funding

This work was funded by NIH Grant R01-AI125097 and R21-AI157864.

## Conflict of interest

The authors declare that the research was conducted in the absence of any commercial or financial relationships that could be construed as a potential conflict of interest.

## Publisher’s note

All claims expressed in this article are solely those of the authors and do not necessarily represent those of their affiliated organizations, or those of the publisher, the editors and the reviewers. Any product that may be evaluated in this article, or claim that may be made by its manufacturer, is not guaranteed or endorsed by the publisher.
